# Modification of termination of resuscitation rule with compression time interval in South Korea

**DOI:** 10.1038/s41598-023-28789-5

**Published:** 2023-01-25

**Authors:** Song Yi Park, Daesung Lim, Ji Ho Ryu, Yong Hwan Kim, Byungho Choi, Sun Hyu Kim

**Affiliations:** 1 Department of Emergency Medicine, Dong-A University College of Medicine, Dong-A University Hospital, Busan, 49201 South Korea; 2 Department of Emergency Medicine, Seoul Medical Center, Seoul, 02053 South Korea; 3 Department of Emergency Medicine, Pusan National University College of Medicine, Pusan National University Yangsan Hospital, Busan, 50612 South Korea; 4 Department of Emergency Medicine, Samsung Changwon Hospital, Sungkyunkwan University School of Medicine, Changwon, 51353 South Korea; 5 Department of Emergency Medicine, University of Ulsan College of Medicine, Ulsan University Hospital, Ulsan, 44033 South Korea

**Keywords:** Cardiology, Health care

## Abstract

This study aimed to validate the predictive performance of the termination of resuscitation (TOR) rule and examine the compression time interval (CTI) as a criterion for modifying the rule. This retrospective observational study analyzed adult out-of-hospital cardiac arrest (OHCA) patients attended by emergency medical service (EMS) providers in mixed urban–rural areas in Korea in 2020 and 2021. We evaluated the predictive performance of basic life support (BLS) and the Korean Cardiac Arrest Research Consortium (KoCARC) TOR rule using the false-positive rate (FPR) and positive predictive value (PPV). We modified the age cutoff criterion and examined the CTI as a new criterion. According to the TOR rule, 1827 OHCA patients were classified into two groups. The predictive performance of the BLS TOR rule had an FPR of 11.7% (95% confidence interval (CI): 5.9–17.5) and PPV of 98.4% (97.6–99.2) for mortality, and an FPR of 3.6% (0.0–7.8) and PPV of 78.6% (75.9–81.3) for poor neurological outcomes at hospital discharge. The predictive performance of the KoCARC TOR rule had an FPR of 5.0% (1.1–8.9) and PPV of 98.9% (98.0–99.8) for mortality, and an FPR of 3.7% (0.0–7.8) and PPV of 50.0% (45.7–54.3) for poor neurological outcomes at hospital discharge. The modified cutoff value for age was 68 years, with an area under the receiver operating characteristic curve over 0.7. In the group that met the BLS TOR rule, the cutoff of the CTI for death was not determined and was 21 min for poor neurological outcomes. In the group that met the KoCARC TOR rule, the cutoff of the CTI for death and poor neurological outcomes at the time of hospital discharge was 25 min and 21 min, respectively. The BLS TOR and KoCARC TOR rules showed inappropriate predictive performance for mortality and poor neurological outcomes. However, the predictive performance of the TOR rule could be supplemented by modifying the age criterion and adding the CTI criterion of the KoCARC.

## Introduction

In the past decades, the global survival rate of out-of-hospital cardiac arrest (OHCA) patients who received cardiopulmonary resuscitation (CPR) has increased to approximately 8.8%^1^. In 2015, the survival rate of OHCA patients in South Korea was 9.3%, and in 2019, it was 9.5% in Seoul^2,3^. The low survival rate and resource allocation of emergency medical services (EMS) raise the issue of termination of resuscitation (TOR) to reduce futile transport to the emergency department (ED). The American Heart Association guidelines for CPR and emergency cardiovascular care suggest the basic life support (BLS) TOR rule for adult OHCA^4^. All three of the following criteria must be present before moving the patient to the ambulance for transport: (1) arrest was not witnessed by an EMS provider or first responder, (2) no return of spontaneous circulation (ROSC) after three full rounds of CPR and automated external defibrillator (AED) analysis, and (3) no AED shocks were delivered before transport.

However, studies from Asian countries to validate the TOR rule reported a high false-positive rate (FPR) and lower positive predictive value (PPV) than reports from North America^5,6^. A study from Japan reported developing their own TOR rule^7^. In Korea, a study by Yoon et al. assessed the predictive performance of the BLS TOR rule for a nationwide cohort in 2019 and reported a high FPR^8^. The study also proposed a new rule called the Korean Cardiac Arrest Research Consortium (KoCARC) TOR rule. It consists of three criteria of the BLS TOR rule and two other criteria, age (> 60 years) and initial pre-hospital rhythm (asystole). However, this study had some limitations. Most of the data were from a high-level ED, and the data demonstrated less possibility of including study populations from rural areas. The time interval, such as the scene time interval, was not considered a criterion due to missing data.

The KoCARC TOR rule was derived from a nationwide cohort, but whether this rule can be applied to a local population was not validated. In particular, the predictive performance of the new rule must be examined in an OHCA population, including a rural area and those transported to a low-level ED, and is considered a limitation. The rule should be validated again in the group described as a limitation because Korea demonstrates a gap in the survival rate of EMS-assessed OHCA patients according to the level of urbanization^9,10^. In Korea’s EMS system, EMS providers cannot declare death at the pre-hospital stage. Emergency physicians sometimes refer to the chest compression period in the pre-hospital stage as the criterion for discontinuing resuscitation efforts. Therefore, this study aimed to validate the predictive performance of the BLS and KoCARC TOR rules and modify the KoCARC TOR rule in adult OHCA patient groups in mixed urban–rural areas in South Korea for emergency physicians.

## Methods

### Study design and setting

This study was a retrospective observational cohort study that analyzed the data of a previous investigation of adult OHCA patients attended by EMS providers in the Busan, Ulsan, Gyeongnam, and Changwon regions in South Korea from November 1, 2019, to January 31, 2020, and from November 1, 2020, to January 31, 2021. We retrospectively evaluated the predictive performance of the BLS and KoCARC TOR rules using FPR and PPV in the study population. We identified the modifiable criteria applicable to the local study population. We examined the time value for the compression time interval (CTI) as a new criterion for the KoCARC TOR rule.

Busan, Ulsan, Gyeongnam, and Changwon regions are located along the coast in the southeastern part of South Korea. Busan and Ulsan are two metropolitan cities, and Changwon is the capital city of Gyeongnam province. The population and area of Busan, Ulsan, Changwon, and Gyeongnam are 3.429 million and 770 km^2^; 1.166 million and 1057 km^2^; 1.07 million and 736.3; and 3.448 million and 10,533 km^2^, respectively. Busan, Ulsan, and Changwon are classified as urban areas, and Gyeongnam as a rural area.

The EMS system in the region, which is government-based and single-tiered, provides basic to intermediate levels of EMS from fire agency headquarters. The EMS resuscitation protocol introduces multiple dispatches (two or more ambulance teams), provides on-site CPR, and transports patients to an ED in an ambulance with ongoing CPR^11^. EMS providers cannot stop CPR unless spontaneous circulation returns in the patient, either on-site or during transportation to the ED. Only physicians in hospital EDs can declare death^12^.

Most of the EMS teams in urban areas consist of three EMS providers, usually including one emergency medical technician. However, EMS teams in rural areas often consist of two EMS providers. Most EMS providers possess registered nurse or first/second-grade emergency medical technician certifications. Generally, ambulances staffed with a physician are not available. In the practices of EMS providers, advanced airway management, intravenous access, fluid and drug administration, and withholding/withdrawal of resuscitation must be overseen by medical directors who are mostly emergency physicians in the area. There is one fire agency headquarters in Busan, Ulsan, Changwon, and Gyeongnam, and one medical director council is in charge of the medical oversight of the four regions. According to local EMS guidelines, EMS providers are advised not to exceed 15 min on the scene.

### Study population and data collection

The inclusion criteria were all patients with OHCA attended by EMS providers during the study periods. The exclusion criteria were patients under 18 years of age, those arrested by trauma, intoxication, or drowning, and those with obvious death or valid do-not-resuscitate orders. Patients were excluded if the arrest occurred in health care facility staffed with a physician or in an ambulance.

There was no integrated cardiac arrest registration system in the study regions. Pre-hospital and hospital data were collected and then matched and merged. Pre-hospital data on all EMS dispatches are collected and managed by regional fire agencies electronically from scene-dispatched EMS providers. This study collected anonymous pre-hospital data from the four fire agency headquarters. Hospital data were collected from 76 treating hospital EDs in the study area, from low-level EDs to high-level EDs.

As patient variables, information about age and sex was collected. As bystander variables, information regarding whether the bystander witnessed, whether the bystander performed CPR and whether the bystander applied an AED was collected. As EMS variables, data on initial rhythm at the scene, advanced airway, epinephrine administration by the EMS provider, and the EMS process time interval, consisting of the response time interval, scene time interval, and transport time interval, were collected. EMS response, scene, and transport time intervals were defined as the time elapsed from the call to EMS arrival at the scene, from EMS arrival at the scene to EMS departure from the scene, and from EMS departure from the scene to EMS arrival at the ED, respectively. The CTI was defined as the sum of the scene and transport time intervals when the EMS providers performed chest compression. As hospital variables, information on survival to discharge and favorable neurological outcomes were collected. Survival to discharge was defined as the case in which a patient survived until hospital discharge. Favorable neurological outcome was determined as cerebral performance category (CPC) 1 or 2, among the following categories: CPC 1 (good cerebral performance), CPC 2 (moderate cerebral disability), CPC 3 (severe cerebral disability), CPC 4 (coma or vegetative state), and CPC 5 (dead)^13^.

### Outcome measures

The primary endpoint of this study was the predictive performance (FPR and PPV) of the BLS and KoCARC TOR rules for death, and poor neurological outcomes at hospital discharge of the study population. The secondary endpoint was to identify the modifiable criteria to supplement the KoCARC TOR rule and examine the time cutoff value of CTI as a new criterion of the KoCARC TOR rules.

### Statistical analysis

We performed a descriptive analysis to examine the study population. Continuous variables are presented as the mean and standard deviation (SD) or median and interquartile range (IQR), and categorical variables are presented as frequencies and proportions. To compare the two groups (the KoCARC TOR rule unmet group and met group), we conducted the Student’s t-test or Mann–Whitney test for numerical variables and the Chi-square test or Fisher’s exact tests for categorical variables after testing for normality. We calculated the predictive performance of the KoCARC TOR rule by sensitivity, specificity, positive and negative predictive value (PPV and NPV), and FPR with a 95% confidence interval (CI) for death and poor neurological outcomes at hospital discharge. The criteria for whether the TOR rule was accurate and reliable were defined when the FPR was close to 0% (less than 1%) with a narrow 95% CI, and the PPV was higher than 99.0%^14,15^. There are three subtypes of the KoCARC TOR rule, depending on the combination of criteria: (1) KoCARC TOR rule I includes the BLS TOR rule and asystole as an initial pre-hospital rhythm; (2) KoCARC TOR rule II includes the BLS TOR rule and age over 60 years; and (3) KoCARC TOR rule III includes the BLS TOR rule, asystole as an initial pre-hospital rhythm, and age over 60 years. The KoCARC TOR rule III is known as the KoCARC TOR rule.

We verified all four rules in the study population. We constructed a receiver operating characteristic (ROC) curve and calculated the area under the ROC curve (AUC) for all the continuous variables to determine their discriminating performance for death and poor neurological outcomes. The optimal cutoff value was determined using the Youden index, and variables with an AUC over 0.7 were considered modifiable criteria. We performed a sensitivity analysis of the predictive performance to determine the CTI cutoff value. All statistical analyses were performed using MedCalc^®^ Statistical Software version 20.027 (MedCalc Software Ltd, Ostend, Belgium; https://www.medcalc.org; 2022). A two-sided *P*-value of < 0.05 was considered statistically significant.

### Ethics statement

The present study protocol was reviewed and approved by the Institutional Review Board of Dong-A University Hospital (approval No. DAUHIRB-EXP-22-045). Informed consent was waived because the present study was a retrospective analysis of existing data that did not contain personal patient information when the data were collected. All methods were conducted in compliance with relevant guidelines and regulations outlined in the Declaration of Helsinki.

## Results

**Figure 1:**
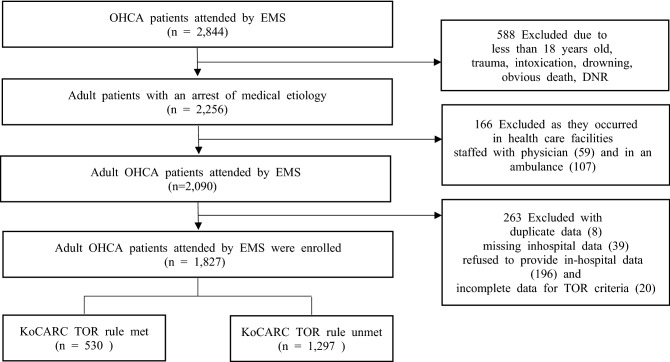
Definition of the study cohort. OHCA, out-of-hospital cardiac arrest; EMS, emergency medical service; KoCARC, Korean Cardiac Arrest Research Consortium; TOR, termination of resuscitation; DNR, do not resuscitate.

A total of 2884 OHCA patients were eligible during the study period. Of these, 588 patients were excluded based on the exclusion criteria, and 59 patients were excluded as cases in healthcare facilities with physicians. Two hundred sixty-three more patients were excluded due to duplicate data, missing in-hospital data, refusal to provide hospital data and incomplete data for TOR criteria. Finally, 1827 patients were categorized into two groups: patients who met all criteria of the KoCARC TOR rule (n = 530) and those who did not (n = 1297) (Fig. 1).

The demographics, pre-hospital characteristics, and hospital outcomes of the study population are presented in Table 1. The mean age of the total study population was 70.8 years, and 63.3% were male. There was no significant difference in the proportion of urban and rural patients between the two groups (*P* = 0.38).

**Table 1:** Demographics, pre-hospital characteristics, and hospital outcomes of the study population.

Characteristics	Total (n = 1827)	KoCARC TOR (−) (n = 1297)	KoCARC TOR (+) (n = 530)	*P*-value
**Patient variables**				
Age^a^	70.8 (15.0)	67.8 (16.1)	78.0 (8.2)	< 0.01
Age^b^	74.0 (61.0–82.0)	70.0 (57.0–81.0)	78.0 (73.0–84.0)	< 0.01
Sex (male)	1156 (63.3)	845 (65.2)	311 (58.7)	0.01
Urban (Busan, Ulsan, Changwon)	1217 (66.6)	856 (66.0)	361 (68.1)	0.38
Rural (Gyeongnam)	610 (33.4)	441 (34.0)	169 (31.9)	
Bystander variables				
Bystander-witnessed (n = 1698)	696 (38.1)	696 (53.7)	0 (0.0)	< 0.01
Bystander CPR (n = 1738)	1016 (55.6)	735 (56.7)	159 (30.0)	0.37
Bystander AED use (n = 1013)	44 (2.4)	45 (3.5)	0 (0.0)	< 0.01
**EMS variables**				
Initial rhythm (n = 1826)				
VF/pulseless VT	245 (13.4)	245 (18.9)	0 (0.0)	< 0.01
PEA	352 (19.3)	352 (27.1)	0 (0.0)	
Asystole	1193 (65.3)	663 (51.1)	530 (100.0)	
Unknown	36 (2.0)	36 (2.8)	0 (0.0)	
Advanced airway (n = 1628)	1408 (77.1)	977 (75.3)	431 (81.3)	0.01
Epinephrine administration (n = 1822)	336 (18.4)	230 (17.7)	106 (20.0)	0.29
EMS process time interval (min)^b^				
EMS response time	7 (6–11)	7 (6–10)	7 (5–11)	0.96
EMS scene time	14 (11–18)	14 (11–18)	14 (11–18)	0.73
EMS transport time	6 (4–10)	6 (4–10)	6 (4–10)	0.46
**KoCARC TOR rule**				
Not witnessed	1002 (54.8)	472 (36.4)	530 (100.0)	
No shock was delivered	1630 (89.2)	1100 (84.8)	530 (100.0)	
No ROSC before transport	1665 (91.1)	1135 (87.5)	530 (100.0)	
Age > 60 years	1392 (76.2)	862 (66.5)	530 (100.0)	
Asystole pre-hospital	1193 (65.3)	663 (51.1)	530 (100.0)	
**Hospital outcomes**				
Survival to hospital discharge	120 (6.6)	114 (8.8)	6 (1.1)	< 0.01
CPC 1, 2 at hospital discharge	83 (4.5)	80 (6.2)	3 (0.6)	< 0.01

KoCARC, Korean Cardiac Arrest Research Consortium; TOR, termination of resuscitation; CPR, cardiopulmonary resuscitation; AED, automated external defibrillator; EMS, emergency medical service; VF, ventricular fibrillation; VT, ventricular tachycardia; PEA, pulseless electrical activity; ROSC, return of spontaneous circulation; CPC, cerebral performance category.

Variables are presented as the mean ± standard deviation^a^, median (quartile 1-quartile 3)^b^, and number (%).

### Predictive performance of the BLS and KoCARC TOR rules for death and poor neurological outcomes at hospital discharge

The predictive performance of the TOR rules for death and poor neurological outcomes are presented in Table 2. The BLS TOR rule showed the highest FPR (11.7%, 95% CI 5.9–17.5%) in predicting death but the lowest FPR (3.6%, 0.0–7.8%) in predicting poor neurological outcomes at hospital discharge.

**Table 2:** Predictive performance of TOR rules for death and poor neurological outcomes at hospital discharge.

TOR rules	Met	Death			SS^a^	SP^a^	FPR^a^	PPV^a^	NPV^a^
		Total	Survival	Death					
BLS rule	(+)	891	14	877	51.4	88.3	11.7	98.4	11.3
	(−)	936	106	830	(49.0–53.8)	(82.5–94.1)	(5.9–17.5)	(97.6–99.2)	(9.3–13.3)
KoCARC I	(+)	675	7	668	39.1	94.2	5.8	99.0	9.8
	(−)	1152	113	1039	(36.8–41.4)	(90.0–98.4)	(1.6–10.0)	(98.2–99.8)	(8.1–11.5)
KoCARC II	(+)	698	11	687	40.2	90.8	9.2	98.4	9.7
	(−)	1129	109	1020	(37.9–42.5)	(85.6–96.0)	(4.0–14.4)	(97.5–99.3)	(8.0–11.4)
KoCARC III	(+)	530	6	524	30.7	95.0	5.0	98.9	8.8
	(−)	1297	114	1183	(28.5–32.9)	(91.1–98.9)	(1.1–8.9)	(98.0–99.8)	(7.3–10.3)

	Met	Poor neurological outcome			SS^a^	SP^a^	FPR^a^	PPV^a^	NPV^a^
		Total	CPC 1,2	CPC 3,4,5					
BLS rule	(+)	891	3	11	30.6	96.4	3.6	78.6	76.4
	(−)	936	81	25	(14.1–42.3)	(92.2–100.0)	(0.0–7.8)	(75.9–81.3)	(70.8–76.4)
KoCARC I	(+)	675	3	4	10.3	96.3	3.7	57.1	69.0
	(−)	1152	78	35	(8.0–19.8)	(92.2–100.0)	(0.0–7.8)	(53.4–60.8)	(66.3–71.7)
KoCARC II	(+)	698	3	8	20.5	96.3	3.7	72.7	71.6
	(−)	1129	78	31	(7.8–33.2)	(92.2–100.0)	(0.0–7.8)	(69.4–76.0)	(69.0–74.2)
KoCARC III	(+)	530	3	3	7.7	96.3	3.7	50.0	68.4
	(−)	1297	78	36	(0.0–16.1)	(92.2–100.0)	(0.0–7.8)	(45.7–54.3)	(65.9–70.9)

TOR, termination of resuscitation; SS, sensitivity; SP, specificity; FPR, false-positive rate; PPV, positive predictive value; NPV, negative predictive value; BLS, basic life support; KoCARC, Korean Cardiac Arrest Research Consortium; CPC, cerebral performance category. CPC 1 and 2 were defined as favorable neurological outcomes, and CPC 3, 4, and 5 as poor neurological outcomes.

^a^Variables are presented as the number and 95% confidence interval.

### Modification of the BLS and KoCARC TOR rules for death and poor neurological outcomes at hospital discharge, incorporating age and compression time interval

**Figure 2:**
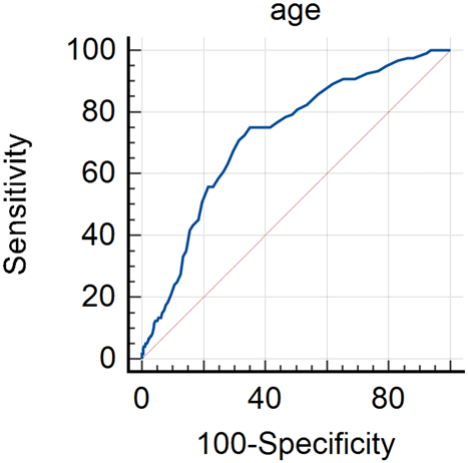
Receiver operating characteristic curve of age for predicting death at hospital discharge. In this figure, the optimal cutoff value of age for predicting survival was over 68 years with a sensitivity of 64.9%, specificity of 75.0%, AUC of 0.723, P-value of < 0.001), and 95% CI of 0.701 to 0.743). AUC, the area under the curve; CI, confidence interval.

In the continuous variables, only age had an AUC over 0.7, and the cutoff value was over 68 years (Fig. 2). In the group that met the BLS TOR rule, the CTI cutoff for death was not determined due to its high FPR, but the cutoff for poor neurological outcomes at hospital discharge with less than 1% of the FPR was 21 min (Table 3). In the group that met the KoCARC TOR rule, the CTI cutoff for death and poor neurological outcomes at hospital discharge with less than 1% of the FPR was 25 min and 21 min, respectively (Table 4). When the additional criteria of age over 68 years and the CTI were applied to the study group, no patient for whom the rule predicted the discontinuation of resuscitation survived (Table 5).

**Table 3:** The cutoff compression time interval in the group met the BLS TOR rule and its predictive performance for death and poor neurological outcomes at hospital discharge.

CTI	Predictive performance for death						Predictive performance for poor neurological outcomes					
	Survival	SS	SP	FPR	PPV	NPV	CPC 1,2	SS	SP	FPR	PPV	NPV
0 ≤	14	51.38	88.33	11.67	98.43	11.32	3	50.9	96.3	3.7	99.7	8.3
1 ≤	14	51.38	88.33	11.67	98.43	11.32	3	50.9	96.3	3.7	99.7	8.3
2 ≤	14	51.38	88.33	11.67	98.43	11.32	3	50.9	96.3	3.7	99.7	8.3
3 ≤	14	51.38	88.33	11.67	98.43	11.32	3	50.9	96.3	3.7	99.7	8.3
4 ≤	14	51.38	88.33	11.67	98.43	11.32	3	50.9	96.3	3.7	99.7	8.3
5 ≤	14	51.38	88.33	11.67	98.43	11.32	3	50.9	96.3	3.7	99.7	8.3
6 ≤	14	51.38	88.33	11.67	98.43	11.32	3	50.9	96.3	3.7	99.7	8.3
7 ≤	14	51.38	88.33	11.67	98.43	11.32	3	50.9	96.3	3.7	99.7	8.3
8 ≤	14	51.38	88.33	11.67	98.43	11.32	3	50.9	96.3	3.7	99.7	8.3
9 ≤	14	51.35	88.24	11.76	98.43	11.22	3	50.8	96.3	3.8	99.7	8.2
10 ≤	14	51.35	88.24	11.76	98.43	11.22	3	50.8	96.3	3.8	99.7	8.2
11 ≤	14	51.26	87.93	12.07	98.43	10.90	3	50.7	96.1	3.9	99.7	7.9
12 ≤	14	51.26	87.93	12.07	98.43	10.90	3	50.7	96.1	3.9	99.7	7.9
13 ≤	13	51.26	88.60	11.40	98.54	10.79	3	50.7	96.1	3.9	99.7	7.8
14 ≤	13	51.20	88.39	11.61	98.54	10.58	3	50.7	95.9	4.1	99.7	7.6
15 ≤	13	50.96	87.50	12.50	98.54	9.72	3	50.5	95.7	4.3	99.7	7.2
16 ≤	12	50.84	87.76	12.24	98.65	9.19	2	50.4	96.9	3.1	99.8	6.6
17 ≤	12	50.72	87.23	12.77	98.65	8.76	2	50.3	96.7	3.3	99.8	6.3
18 ≤	12	50.55	86.36	13.64	98.65	8.12	2	50.2	96.4	3.6	99.8	5.7
19 ≤	10	50.55	88.10	11.90	98.88	7.91	2	50.2	96.4	3.6	99.8	5.7
20 ≤	8	50.46	89.61	10.39	99.10	7.37	1	50.1	98.0	2.0	99.9	5.2
21 ≤	7	50.37	90.28	9.72	99.21	6.94	0	50.0	100.0	0.0	100.0	4.9
22 ≤	7	50.28	89.86	10.14	99.21	6.62	0	50.0	100.0	0.0	100.0	4.8
23 ≤	6	50.23	90.77	9.23	99.33	6.30	0	49.9	100.0	0.0	100.0	4.6
24 ≤	6	50.11	90.16	9.84	99.33	5.88	0	49.9	100.0	0.0	100.0	4.3
25 ≤	4	50.00	92.45	7.55	99.55	5.24	0	49.7	100.0	0.0	100.0	3.7
26 ≤	4	49.83	91.49	8.51	99.55	4.59	0	49.6	100.0	0.0	100.0	3.3
27 ≤	4	49.83	91.49	8.51	99.55	4.59	0	49.6	100.0	0.0	100.0	3.3
28 ≤	2	49.83	95.35	4.65	99.78	4.38	0	49.6	100.0	0.0	100.0	3.3
29 ≤	2	49.72	94.87	5.13	99.78	3.95	0	49.5	100.0	0.0	100.0	2.9
30 ≤	2	49.66	94.59	5.41	99.78	3.74	0	49.4	100.0	0.0	100.0	2.7
31 ≤	2	49.55	93.94	6.06	99.78	3.31	0	49.3	100.0	0.0	100.0	2.2
32 ≤	1	49.53	96.67	3.33	99.89	3.10	0	49.3	100.0	0.0	100.0	2.0
33 ≤	1	49.44	96.30	3.70	99.89	2.78	0	49.2	100.0	0.0	100.0	1.7
34 ≤	1	49.33	95.65	4.35	99.89	2.35	0	49.1	100.0	0.0	100.0	1.5
35 ≤	1	49.33	95.65	4.35	99.89	2.35	0	49.1	100.0	0.0	100.0	1.5
36 ≤	1	49.33	95.65	4.35	99.89	2.35	0	49.1	100.0	0.0	100.0	1.5
37 ≤	1	49.33	95.65	4.35	99.89	2.35	0	49.1	100.0	0.0	100.0	1.5
38 ≤	1	49.23	94.74	5.26	99.89	1.92	0	49.1	100.0	0.0	100.0	1.3
39 ≤	1	49.17	94.12	5.88	99.89	1.71	0	49.0	100.0	0.0	100.0	1.1
40 ≤	1	49.14	93.75	6.25	99.89	1.60	0	49.0	100.0	0.0	100.0	1.0
41 ≤	1	49.04	91.67	8.33	99.89	1.18	0	49.0	100.0	0.0	100.0	0.7
42 ≤	1	49.01	90.91	9.09	99.89	1.07	0	48.9	100.0	0.0	100.0	0.6
43 ≤	1	49.01	90.91	9.09	99.89	1.07	0	48.9	100.0	0.0	100.0	0.6
44 ≤	1	49.01	90.91	9.09	99.89	1.07	0	48.9	100.0	0.0	100.0	0.6
45 ≤	1	49.01	90.91	9.09	99.89	1.07	0	48.9	100.0	0.0	100.0	0.6
46 ≤	1	49.01	90.91	9.09	99.89	1.07	0	48.9	100.0	0.0	100.0	0.6
47 ≤	1	48.95	88.89	11.11	99.89	0.85	0	48.9	100.0	0.0	100.0	0.4

Variables are presented as numbers and percentages (%). The survival number and CPC 1 and 2 indicate patients who met the BLS TOR rule but survived and showed good neurological outcomes. BLS, basic life support; TOR, termination of resuscitation; CTI, compression time interval; SS, sensitivity; SP, specificity; FPR, false-positive rate; PPV, positive predictive value; NPV, negative predictive value.

**Table 4:** The cutoff compression time interval in the group met the KoCARC TOR rule and its predictive performance for death and poor neurological outcomes at hospital discharge.

CTI	Predictive performance for death						Predictive performance for poor neurological outcomes					
	Survival	SS	SP	FPR	PPV	NPV	CPC 1,2	SS	SP	FPR	PPV	NPV
0 ≤	6	30.7	95.0	5.0	98.9	8.8	3	30.2	96.3	3.7	99.4	6.0
1 ≤	6	30.7	95.0	5.0	98.9	8.8	3	30.2	96.3	3.7	99.4	6.0
2 ≤	6	30.7	95.0	5.0	98.9	8.8	3	30.2	96.3	3.7	99.4	6.0
3 ≤	6	30.7	95.0	5.0	98.9	8.8	3	30.2	96.3	3.7	99.4	6.0
4 ≤	6	30.8	95.0	5.0	98.9	8.8	3	30.3	96.3	3.7	99.4	6.0
5 ≤	6	30.8	95.0	5.0	98.9	8.9	3	30.3	96.3	3.7	99.4	6.1
6 ≤	6	30.7	95.0	5.0	98.9	8.9	3	30.2	96.3	3.7	99.4	6.1
7 ≤	6	30.8	95.0	5.0	98.9	8.9	3	30.3	96.3	3.7	99.4	6.1
8 ≤	6	30.8	95.0	5.0	98.8	9.0	3	30.3	96.3	3.7	99.4	6.1
9 ≤	6	30.8	95.0	5.0	98.8	8.9	3	30.3	96.3	3.8	99.4	6.1
10 ≤	6	30.9	95.0	5.0	98.8	9.0	3	30.3	96.3	3.8	99.4	6.1
11 ≤	6	31.0	94.8	5.2	98.8	8.9	3	30.5	96.1	3.9	99.4	6.0
12 ≤	6	31.1	94.8	5.2	98.8	9.0	3	30.6	96.1	3.9	99.4	6.1
13 ≤	6	30.8	94.7	5.3	98.8	9.1	3	30.3	96.1	3.9	99.4	6.1
14 ≤	6	31.0	94.6	5.4	98.7	9.3	3	30.5	95.9	4.1	99.4	6.2
15 ≤	6	30.9	94.2	5.8	98.7	9.1	3	30.4	95.7	4.3	99.3	6.2
16 ≤	5	30.7	94.9	5.1	98.8	9.1	2	30.2	96.9	3.1	99.5	6.0
17 ≤	5	30.7	94.7	5.3	98.7	9.1	2	30.1	96.7	3.3	99.5	6.0
18 ≤	5	31.0	94.3	5.7	98.7	9.2	2	30.4	96.4	3.6	99.5	5.8
19 ≤	4	30.8	95.2	4.8	98.8	9.5	2	30.2	96.4	3.6	99.4	6.3
20 ≤	2	31.3	97.4	2.6	99.4	9.8	1	30.6	98.0	2.0	99.7	6.4
21 ≤	2	31.0	97.3	2.7	99.3	9.9	0	30.3	100.0	0.0	100.0	6.4
22 ≤	1	30.4	98.6	1.4	99.6	10.4	0	29.7	100.0	0.0	100.0	6.9
23 ≤	1	30.8	98.5	1.5	99.6	10.8	0	30.1	100.0	0.0	100.0	7.3
24 ≤	1	30.6	98.4	1.6	99.5	11.1	0	29.8	100.0	0.0	100.0	7.4
25 ≤	0	29.1	100.0	0.0	100.0	11.0	0	28.3	100.0	0.0	100.0	7.2
26 ≤	0	28.9	100.0	0.0	100.0	11.2	0	28.0	100.0	0.0	100.0	7.4
27 ≤	0	28.8	100.0	0.0	100.0	12.8	0	27.8	100.0	0.0	100.0	8.4
28 ≤	0	28.4	100.0	0.0	100.0	13.0	0	27.5	100.0	0.0	100.0	9.4
29 ≤	0	27.7	100.0	0.0	100.0	13.1	0	26.8	100.0	0.0	100.0	9.1
30 ≤	0	27.1	100.0	0.0	100.0	13.7	0	26.1	100.0	0.0	100.0	9.2

Variables are presented as numbers and percentages. The survival number and CPC 1 and 2 indicate patients who met the KoCARC TOR rule but survived and showed good neurological outcomes. KoCARC, Korean Cardiac Arrest Research Consortium; TOR, termination of resuscitation; CTI, compression time interval; SS, sensitivity; SP, specificity; FPR, false-positive rate; PPV, positive predictive value; NPV, negative predictive value.

**Table 5:** Predictive performance of revised KoCARC TOR rule for death and poor neurological outcomes at hospital discharge.

TOR rules	Met	Death			SS^a^	SP^a^	FPR^a^	PPV^a^	NPV^a^
		Total	Survival	Death					
Revised KoCARC III	(+)	157	0	157	9.2	100.0	0.0	100.0	7.2
	(−)	1670	120	1550	(7.9–10.5)	–	–	–	(6.0–8.4)

	Met	Poor neurological outcomes			SS^a^	SP^a^	FPR^a^	PPV^a^	NPV^a^
		Total	CPC 1,2	CPC 3,4,5					
Revised KoCARC III	(+)	530	3	3	80.9	100.0	0.0	100.0	69.2
	(−)	1297	78	36	(79.1–82.7)	–	–	–	(67.1–71.3)

TOR, termination of resuscitation; SS, sensitivity; SP, specificity; FPR, false-positive rate; PPV, positive predictive value; NPV, negative predictive value; BLS, basic life support; KoCARC, Korean Cardiac Arrest Research Consortium; CPC, cerebral performance category. CPC 1 and 2 were defined as favorable neurological outcomes, and CPC 3, 4, and 5 as poor neurological outcomes.

^a^Variables are presented as the number and 95% confidence interval.

## Discussion

This study aimed to validate whether the BLS and KoCARC TOR rules could be used in a local cohort and identify the modifiable criterion and CTI cutoff for modifying the TOR rule in adult OHCA patient groups in mixed urban–rural areas in Korea. The predictive performance of all the TOR rules was not identified as an appropriate decision-making tool to terminate the resuscitation effort due to the high FPR. Age was the only modifiable criterion, and the cutoff was over 68 years old. The CTI cutoff for mortality was 25 min in the group that met the KoCARC TOR criterion, and for poor neurological outcomes, it was 21 min in both groups. This study demonstrated strength in verifying its applicability and reliability by reapplying the KoCARC TOR rule derived from a national cohort to a local patient group.

The BLS TOR rule showed a relatively low predictive performance with the highest FPR in this study. This finding is consistent with other studies that reported the predictive performance of the BLS TOR rule in Korea. A study using data from Seoul reported 69.6% specificity and 95.9% PPV for predicting death^16^. In another study in Daegu, located in the middle of South Korea, the BLS TOR rule showed 85% specificity and 99% PPV for predicting unfavorable survival outcomes^17^. In contrast, studies from North America reported more than 99% PPV and less than 1% FPR for a clinical prediction of the BLS TOR rule^18–20^. This gap is due to the difference in the EMS system and cultural background. In Korea, resuscitation is rarely withdrawn at the pre-hospital stage, and almost all OHCA patients are transported to the ED. It is rare for patients to clarify their intention to do-not-resuscitation in advance, although it has improved since the enforcement of the Life-sustaining Treatment Decision Act in 2018^21,22^.

The age gap in both the study populations is hypothesized as the main reason for the poor performance of the KoCARC rule. Our study population was older (70.8 ± 15.0 years) than that in the KoCARC study (67.43 ± 15.80 years). In the KoCARC study, the cutoff value for the age criterion was over 60 years, whereas it was 68 years older in our research (in the ROC curve). However, when we changed the age criterion to 68 years, the FPR was 1.7% (1.1–2.3), and the PPV was 99.6% (99.3–99.9), indicating that the values were still inappropriate. The TOR rule should minimize the inclusion of potential survivors in the rule. However, considering our study's results, the age criterion's cutoff value seemed to vary depending on the study population. In addition, estimating the patient’s age only by appearance may be inaccurate at the pre-hospital and hospital stages. Moreover, there are many cases where physiological and chronological age is not matched. Thus, age should be approached carefully as a criterion for discontinuing resuscitation efforts.

It can be assumed that our study group was older than the KoCARC group because it included patients from rural areas. However, this assumption was not true. In the group that met the KoCARC TOR rule, the proportion of patients in rural areas was 31.9%, which was less than that in the group in which the KoCARC TOR rule was not met (34.0%). There was no significant difference in the proportion of patients in rural areas in the two groups. Actually, in this study, the mean ± SD and median age and IQR of the patients were 70.8 (± 15.0) and 74 (61.0–81.0) years in urban areas and 71.8 (± 14.9) and 76.0 (61.0–83.0) years in rural areas, respectively. There was no significant difference in age (*P* = 0.132). Further research is needed to prove if any insignificant difference in the age of OHCA patients in urban and rural areas was limited to this study area or is similar in other areas.

In this study, the CTI was added to supplement the predictive performance of the TOR rules. However, we could not derive the CTI cutoff value for the BLS TOR rule that predicted mortality as no improvement in the FPR was observed. The probable reason could be the high FPR of the BLS TOR rule. In this scenario, the BLS TOR rule was identified as inappropriate for decision-making by either EMS providers at the pre-hospital stage or emergency physicians in the ED. Kim et al. reported using the STI as an indicator of TOR in Seoul, Korea, in 2015^14^. However, we could not apply the STI as the criterion of the TOR rule for this study population. The local guidelines for medical direction recommend that the STI should not exceed 15 min^23^. The CTI was the actual period when the EMS providers performed chest compression and ventilation. There may be cases where bystander CPR was either performed or not. However, we did not consider this point because even if bystander CPR was performed, the quality of CPR could not always be guaranteed. In some cases, the EMS processing time interval may affect decision-making for discontinuing the resuscitation effort. Accordingly, we analyzed the processing time interval cutoff value for death and poor neurological outcomes at hospital discharge, which was 42 min and 28 min, respectively (Supplementary 1).

Several limitations of our study must be acknowledged. First, we validated the BLS TOR rule in our research. Still, in some patients, advanced cardiovascular life support (ACLS) was performed using epinephrine at the pre-hospital stage as part of a national pilot project. However, we included this study population because local emergency physicians may use the BLS TOR (terminate resuscitation) rule to decide when to stop resuscitation efforts, even if the patient received epinephrine in the pre-hospital setting. This is because the pre-hospital ACLS TOR rule has yet to be established in Korea. Moreover, this is not standard ACLS, and epinephrine administration did not improve the survival rate in our previous study^24^. Thus, no significant impact on the study findings is expected. Second, we did not consider in-hospital resuscitation time and interventions, such as coronary reperfusion therapy and targeted temperature management. Moreover, many emergency physicians persist on ACLS for at least 20 min in OHCA patients regarding the in-hospital resuscitation time. Still, we have yet to verify whether this aspect was maintained even in low-level EDs. Third, the CTI criterion was added to the KoCARC TOR rule. Decision-making rules should be as simple as possible to apply. Subsequent studies on the feasibility and reliability of the revised TOR rule are needed.

In conclusion, the BLS and KoCARC TOR rules showed inappropriate predictive performance with high FPR for death and poor neurological outcomes at hospital discharge in adult OHCA patients from mixed urban–rural areas in South Korea. However, it is proposed that the predictive performance could be supplemented by modifying the age criterion of the KoCARC TOR rule from 60 to 68 years and adding the CTI cutoff criterion of 25 min for death and 21 min for poor neurological outcomes. This modified TOR rule was derived retrospectively, and further studies are needed to determine whether this TOR rule could be used in actual patients.

## Supplementary Information

Supplementary Information.

## Data Availability

Raw data were generated at national fire agencies in Korea. The derived data supporting the findings of this study are available from the corresponding author upon reasonable request.

## Abbreviations

AED: Automated external defibrillator
BLS: Basic life support
CI: Confidence interval
CPR: Cardiopulmonary resuscitation
CTI: Compression time interval
ED: Emergency department
EMS: Emergency medical service
FPR: False-positive rate
IQR: Interquartile range
SD: Standard deviation
TOR: Termination of resuscitation
KoCARC: Korean Cardiac Arrest Research Consortium
OHCA: Out-of-hospital cardiac arrest
PPV: Positive predictive value
ROSC: Return of spontaneous circulation
