# Supplementary material for: Modification of termination of resuscitation rule with compression time interval in South Korea
Source: Sci Rep. 2023 Jan 25;13:1403. doi: 10.1038/s41598-023-28789-5 (PMC9876889; doi:10.1038/s41598-023-28789-5)
Supplement: Supplementary file 1 — Supplementary Information. [file 41598_2023_28789_MOESM1_ESM.docx]

|  | Predictive performance for death | | | | | | Predictive performance for poor neurological outcomes | | | | | |
| --- | --- | --- | --- | --- | --- | --- | --- | --- | --- | --- | --- | --- |
| PTI | Survival | SS | SP | FPR | PPV | NPV | CPC 1,2 | SS | SP | FPR | PPV | NPV |
| 0≤ | 6 | 30.7 | 95.0 | 5.0 | 98.9 | 8.8 | 3 | 30.2 | 96.3 | 3.7 | 99.4 | 6.0 |
| 1≤ | 6 | 30.7 | 95.0 | 5.0 | 98.9 | 8.8 | 3 | 30.2 | 96.3 | 3.7 | 99.4 | 6.0 |
| 2≤ | 6 | 30.7 | 95.0 | 5.0 | 98.9 | 8.8 | 3 | 30.2 | 96.3 | 3.7 | 99.4 | 6.0 |
| 3≤ | 6 | 30.7 | 95.0 | 5.0 | 98.9 | 8.8 | 3 | 30.2 | 96.3 | 3.7 | 99.4 | 6.0 |
| 4≤ | 6 | 30.7 | 95.0 | 5.0 | 98.9 | 8.8 | 3 | 30.2 | 96.3 | 3.7 | 99.4 | 6.0 |
| 5≤ | 6 | 30.7 | 95.0 | 5.0 | 98.9 | 8.8 | 3 | 30.2 | 96.3 | 3.7 | 99.4 | 6.0 |
| 6≤ | 6 | 30.7 | 95.0 | 5.0 | 98.9 | 8.8 | 3 | 30.2 | 96.3 | 3.7 | 99.4 | 6.0 |
| 7≤ | 6 | 30.7 | 95.0 | 5.0 | 98.9 | 8.8 | 3 | 30.2 | 96.3 | 3.7 | 99.4 | 6.0 |
| 8≤ | 6 | 30.7 | 95.0 | 5.0 | 98.9 | 8.8 | 3 | 30.2 | 96.3 | 3.7 | 99.4 | 6.0 |
| 9≤ | 6 | 30.7 | 95.0 | 5.0 | 98.9 | 8.8 | 3 | 30.2 | 96.3 | 3.7 | 99.4 | 6.0 |
| 10≤ | 6 | 30.7 | 95.0 | 5.0 | 98.9 | 8.8 | 3 | 30.2 | 96.3 | 3.7 | 99.4 | 6.0 |
| 11≤ | 6 | 30.7 | 95.0 | 5.0 | 98.9 | 8.8 | 3 | 30.2 | 96.3 | 3.7 | 99.4 | 6.0 |
| 12≤ | 6 | 30.7 | 95.0 | 5.0 | 98.9 | 8.8 | 3 | 30.2 | 96.3 | 3.7 | 99.4 | 6.0 |
| 13≤ | 6 | 30.7 | 95.0 | 5.0 | 98.9 | 8.8 | 3 | 30.2 | 96.3 | 3.7 | 99.4 | 6.0 |
| 14≤ | 6 | 30.7 | 95.0 | 5.0 | 98.9 | 8.7 | 3 | 30.3 | 96.3 | 3.8 | 99.4 | 6.0 |
| 15≤ | 6 | 30.7 | 94.9 | 5.1 | 98.9 | 8.6 | 3 | 30.4 | 96.2 | 3.8 | 99.4 | 5.9 |
| 16≤ | 6 | 30.6 | 94.8 | 5.2 | 98.9 | 8.5 | 3 | 30.4 | 96.1 | 3.9 | 99.4 | 5.8 |
| 17≤ | 6 | 30.6 | 94.8 | 5.2 | 98.9 | 8.5 | 3 | 30.3 | 96.1 | 3.9 | 99.4 | 5.9 |
| 18≤ | 6 | 30.6 | 94.7 | 5.3 | 98.9 | 8.3 | 3 | 30.6 | 96.0 | 4.0 | 99.4 | 5.8 |
| 19≤ | 6 | 30.6 | 94.6 | 5.4 | 98.9 | 8.2 | 3 | 30.6 | 95.9 | 4.1 | 99.4 | 5.9 |
| 20≤ | 5 | 30.5 | 95.4 | 4.6 | 99.1 | 7.9 | 2 | 30.5 | 97.2 | 2.8 | 99.6 | 5.9 |
| 21≤ | 5 | 30.5 | 95.2 | 4.8 | 99.1 | 7.7 | 2 | 30.4 | 97.1 | 2.9 | 99.6 | 5.8 |
| 22≤ | 5 | 30.5 | 95.1 | 4.9 | 99.1 | 7.6 | 2 | 30.4 | 97.0 | 3.0 | 99.6 | 5.9 |
| 23≤ | 5 | 30.4 | 94.9 | 5.1 | 99.1 | 7.2 | 2 | 30.9 | 96.9 | 3.1 | 99.5 | 6.0 |
| 24≤ | 4 | 30.3 | 95.6 | 4.4 | 99.2 | 6.6 | 1 | 30.8 | 98.3 | 1.7 | 99.8 | 5.9 |
| 25≤ | 2 | 30.3 | 97.6 | 2.4 | 99.6 | 6.4 | 1 | 30.1 | 98.2 | 1.8 | 99.7 | 5.9 |
| 26≤ | 2 | 30.3 | 97.6 | 2.4 | 99.6 | 6.4 | 1 | 29.9 | 98.2 | 1.8 | 99.7 | 6.2 |
| 27≤ | 2 | 30.2 | 97.5 | 2.5 | 99.6 | 5.9 | 1 | 30.0 | 98.1 | 1.9 | 99.7 | 6.2 |
| 28≤ | 1 | 30.2 | 98.7 | 1.3 | 99.8 | 5.7 | 0 | 29.9 | 100.0 | 0.0 | 100.0 | 6.3 |
| 29≤ | 1 | 30.1 | 98.6 | 1.4 | 99.8 | 5.4 | 0 | 30.5 | 100.0 | 0.0 | 100.0 | 6.5 |
| 30≤ | 1 | 30.0 | 98.5 | 1.5 | 99.8 | 4.9 | 0 | 29.8 | 100.0 | 0.0 | 100.0 | 6.3 |
| 31≤ | 1 | 30.0 | 98.4 | 1.6 | 99.8 | 4.7 | 0 | 29.7 | 100.0 | 0.0 | 100.0 | 6.4 |
| 32≤ | 1 | 29.9 | 98.3 | 1.7 | 99.8 | 4.4 | 0 | 28.5 | 100.0 | 0.0 | 100.0 | 6.6 |
| 33≤ | 1 | 29.8 | 98.1 | 1.9 | 99.8 | 3.9 | 0 | 28.4 | 100.0 | 0.0 | 100.0 | 6.5 |
| 34≤ | 1 | 29.8 | 98.0 | 2.0 | 99.8 | 3.9 | 0 | 29.7 | 100.0 | 0.0 | 100.0 | 7.0 |
| 35≤ | 1 | 29.7 | 97.8 | 2.2 | 99.8 | 3.5 | 0 | 28.8 | 100.0 | 0.0 | 100.0 | 6.7 |
| 36≤ | 1 | 29.7 | 97.7 | 2.3 | 99.8 | 3.2 | 0 | 28.2 | 100.0 | 0.0 | 100.0 | 7.5 |
| 37≤ | 1 | 29.6 | 97.5 | 2.5 | 99.8 | 3.0 | 0 | 29.2 | 100.0 | 0.0 | 100.0 | 7.6 |
| 38≤ | 1 | 29.6 | 97.4 | 2.6 | 99.8 | 2.9 | 0 | 29.2 | 100.0 | 0.0 | 100.0 | 8.4 |
| 39≤ | 1 | 29.6 | 97.3 | 2.7 | 99.8 | 2.8 | 0 | 28.9 | 100.0 | 0.0 | 100.0 | 8.4 |
| 40≤ | 1 | 29.5 | 97.2 | 2.8 | 99.8 | 2.7 | 0 | 27.7 | 100.0 | 0.0 | 100.0 | 8.9 |
| 41≤ | 1 | 29.5 | 96.9 | 3.1 | 99.8 | 2.4 | 0 | 27.2 | 100.0 | 0.0 | 100.0 | 8.2 |
| 42≤ | 0 | 29.5 | 100.0 | 0.0 | 100.0 | 2.2 | 0 | 27.2 | 100.0 | 0.0 | 100.0 | 8.1 |
| 43≤ | 0 | 29.4 | 100.0 | 0.0 | 100.0 | 2.0 | 0 | 25.3 | 100.0 | 0.0 | 100.0 | 7.8 |
| 44≤ | 0 | 29.4 | 100.0 | 0.0 | 100.0 | 1.9 | 0 | 25.4 | 100.0 | 0.0 | 100.0 | 7.6 |
| 45≤ | 0 | 29.3 | 100.0 | 0.0 | 100.0 | 1.6 | 0 | 23.9 | 100.0 | 0.0 | 100.0 | 6.6 |

**Supplementary**. The cutoff process time interval in the group met the KoCARC TOR rule and its predictive performance for death and poor neurological outcomes at hospital discharge. Variables are presented as numbers and percentages (%). The survival number and CPC 1 and 2 indicate patients who met the KoCARC TOR rule but survived and showed good neurological outcomes. KoCARC, Korean Cardiac Arrest Research Consortium; TOR, termination of resuscitation; CTI, compression time interval; SS, sensitivity; SP, specificity; FPR, false-positive rate; PPV, positive predictive value; NPV, negative predictive value
